# Quantitative evaluation of *RASSF1A *methylation in the non-lesional, regenerative and neoplastic liver

**DOI:** 10.1186/1471-2407-6-89

**Published:** 2006-04-10

**Authors:** Sonia Di Gioia, Paolo Bianchi, Annarita Destro, Fabio Grizzi, Alberto Malesci, Luigi Laghi, Massimo Levrero, Alberto Morabito, Massimo Roncalli

**Affiliations:** 1 Laboratory of Molecular Genetics, Humanitas Clinical Institute of Rozzano, Milan, Italy; 2 Scientific Direction, Humanitas Clinical Institute of Rozzano, Milan, Italy; 3 Department of Gastroenterology, Humanitas Clinical Institute of Rozzano, Milan, Italy; 4 Department of Pathology, Humanitas Clinical Institute of Rozzano, Milan, Italy; 5 Laboratory of Gene Expression, Fondazione Andrea Cesalpino, University of Rome La Sapienza, Rome, Italy; 6 University of Milan. Milan, Italy

## Abstract

**Background:**

Epigenetic changes during ageing and their relationship with cancer are under the focus of intense research. *RASSF1A *and *NORE1A *are novel genes acting in concert in the proapoptotic pathway of the RAS signalling. While *NORE1A *has not been previously investigated in the human liver, recent reports have suggested that *RASSF1A *is frequently epigenetically methylated not only in HCC but also in the cirrhotic liver.

**Methods:**

To address whether epigenetic changes take place in connection to age and/or to the underlying disease, we investigated *RASSF1A *and *NORE1A *gene promoter methylation by conventional methylation specific PCR and Real-Time MSP in a series of hepatitic and non-hepatitic livers harboring regenerative/hyperplastic (cirrhosis/focal nodular hyperplasia), dysplastic (large regenerative, low and high grade dysplastic nodules) and neoplastic (hepatocellular adenoma and carcinoma) growths.

**Results:**

In the hepatitic liver (chronic hepatitic/cirrhosis, hepatocellular nodules and HCC) we found widespread *RASSF1A *gene promoter methylation with a methylation index that increased from regenerative conditions (cirrhosis) to hepatocellular nodules (p < 0.01) to HCC (p < 0.001). In the non-hepatitic liver a consistent pattern of gene methylation was also found in both lesional (focal nodular hyperplasia and hepatocellular adenoma) and non-lesional tissue. Specifically, hepatocellular adenomas (HA) showed a methylation index significantly higher than that detected in focal nodular hyperplasia (FNH) (p < 0.01) and in non-lesional tissue (p < 0.001). In non-lesional liver also the methylation index gradually increased by ageing (p = 0.002), suggesting a progressive spreading of methylated cells over time. As opposed to *RASSF1A *gene promoter methylation, *NORE1A *gene was never found epigenetically alterated in both hepatitic and non-hepatitic liver.

**Conclusion:**

We have shown that in non-lesional, regenerative and neoplastic liver the *RASSF1A *gene is increasingly methylated, that this condition takes place as an age-related phenomenon and that the early setting and spreading over time of an epigenetically methylated hepatocyte subpopulation, might be related to liver tumorigenesis.

## Background

A new family of tumor suppressor genes encoding RAS-binding proteins and named RASSF1 has been recently discovered [1,2]. They are located within a critical region at 3p21.3 and have two main splicing isoforms *RASSF1A *and *1C*. The expression of the longer isoform, *RASSF1A*, is lost in many tumor lines and primary tumors by promoter methylation, while *RASSF1C *remains unmethylated [2-11]. It has been suggested that *RASSF1A *methylation is one of the most common aberration so far identified in human cancers and that the loss of the functional protein may promote the development of many human tumors [12]. Methylation of *RASSF1A *promoter has been documented in 85% [13], 100% [14], 95% [15], 93% [16] and 67% [17] of hepatocellular carcinomas (HCC). Interestingly, non neoplastic hepatitic/cirrhotic tissue usually adjacent to HCC, beside other methylated genes [18,19], frequently shows *RASSF1A *gene methylation ranging from 70% [13,15] to 82.75% [14], while non-neoplastic liver, or far from tumors, is not methylated [13,15,17]. These results suggest that *RASSF1A *methylation might be a potential marker of incipient malignancy in the human hepatocarcinogenesis.

In an autopsy series, Waki et al recently reported [20], that *RASSF1A *gene promoter is largely methylated in the human liver and that this epigenetic change may be related to age.

Given that age-related methylation is a process that affect non-lesional cells and precedes tumor formation [21], the early setting of a hepatocyte subpopulation with *RASSF1A *methylation, might be a potential and long-lasting for oncogenic injuries.

We designed a study aimed to ascertain the frequency of *RASSF1A *methylation in non-lesional, cirrhotic, hyperplastic, dysplastic and neoplastic liver. Moreover we performed a quantification study, by Real-Time PCR, to document whether the gene is methylated in relation to age and/or to the underlying disease. Given that the functional proapoptotic role of *RASSF1A *as tumor suppressor, has been recently associated to the homologue gene *NORE1A *[22-24] we also evaluated the methylation status of the latter gene in the same non-lesional and pathological conditions.

## Methods

### Tissue samples and DNA extraction

Consecutive cases from 38 hepatitic/cirrhotic patients with and without hepatocellular carcinoma (HCC) and/or hepatocellular nodules (HN), from 13 patients with focal nodular hyperplasia (FNH) or hepatocellular adenoma (HA) and 5 human cell lines (hepatoma cell lines: Plc/Prf/5, Huh6, Huh7 and HepG2, and Chang, a normal hepatocyte cell line) were collected for study. Patient's characteristics (age, sex, etiology of hepatitis, type of lesions) are reported in Table 1. The series included 26 HCCs, 17 high-grade dysplastic nodules (HGDN), 28 non high-grade dysplastic nodules (large regenerative/low-grade dysplastic nodules), 5 hepatocellular adenomas (HA) and 8 focal nodular hyperplasias (FNH). The study was conducted in accordance with the guidelines of the Ethics Committee and Internal Scientific Board of our institution (Istituto Clinico Humanitas, Rozzano, Milan, Italy).

After identification of the different lesions on the hematoxylin-eosin stained slides, individual lesions were carefully microdissected from 20-μm-thick paraffin sections. In all cases hepatocytes represented the main cellular population (≥ 80% of overall cellularity). In selected cases (Table 1) of FNH and HA, different samples were taken from the same lesional and non-lesional liver in order to assess individual inter-sample methylation pattern and levels heterogeneity. The non-lesional liver samples were taken far from the lesions.

Collected materials were dewaxed by washing in xylene, and rinsed in ethanol. The dried tissues were digested using proteinase K and subjected to classical DNA extraction using phenol/chloroform/isoamylalcohol and ethanol precipitation. After precipitation, DNA was resuspended in water and quantified by spectrophotometric analysis (260 nm).

### RASSF1A and NORE1A methylation-specific PCR (MSP)

#### Sodium bisulfite modification

We designed a set of primers targeted to amplify a 111 base pair sequence located in the upstream region of the *RASSF1A *promoter (ranging from -135 to -24 base pairs from the starting site of exon 1) (Table 2). This sequence was selected because it showed the highest CpG island density of the whole promoter.

Methylation-specific PCR was carried-out as previously reported [18,25]. Genomic DNA was modified by treatment with sodium bisulfite, which converts all unmethylated cytosines to uracil, then to thymidine during the subsequent PCR step. One μg of DNA in a volume of 50 μl were denatured by adding sodiumhydroxide to a final concentration of 0.2 M and incubating for 10 min at 37°C. Thirty μl of 10 mM hydroquinone and 520 μl of 3 M sodium bisulfite (pH 5.0), both freshly prepared, were added to the denatured DNA. The sample was mixed gently, overlaid with enough mineral oil to cover the surface of the aqueous phase, and incubated at 55°C for 16–20 h. Under this conditions, complete selective conversion of unmethylated cytosine to uracil can be achieved [26]. The modified DNA was desalted through Wizard DNA Clean-up System (Promega) and eluted in 50 μl of distillated water. Desulphonation was carried-out adding 5.5 μl of NaOH 3 M and incubating DNA for 5 min at room temperature. Modified DNA was precipited with sodium acetated 3 M, ethanol and 1 μl of glycogen as carrier; finally was resuspended in 50 μl of distillated water and DNA concentration was measured by spectrophotometric analysis. The DNA was stored at -20°C until use.

The methylation status of *RASSF1A *and *NORE1A *was determined by the MSP method of Herman et al. with the following modifications, previously described [26]. Five microliters of modified DNA were amplified in a stage I PCR with a primer set that recognise the bisulfite-modified template but do not discriminate between methylated and unmethylated alleles (Table 2). Three microliters of the stage I PCR product were subjected to a stage II PCR in which primers specific to methylated or unmethylated template were used. DyNAzyme polymerase (Finnzymes) and 0.4 μmol/l of each primer in a 30 μl final volume were used in all PCRs. The PCR protocol for stage I was as follows: 94°C for 4 min, then denature at 94°C for 30 sec, anneal at 48°C for 30 sec for *RASSF1A *and 52°C for 30 sec for *NORE1A*, extension at 72°C for 30 sec for 20 cycles, followed by a 10 min of final extension. Primer sequences used in a stage II amplifications are detailed in Table 2. Annealing temperature for *RASSF1A *was 55°C to amplify methylated and unmethylated sequences, for a total of 25 cycles and annealing temperature for *NORE1A *was 58°C to amplify methylated and unmethylated sequences, for a total of 25 cycles. This nested PCRs are able to detect 1 methylated allele in 50,000 unmethylated alleles [26]. All PCRs were performed with positive methylation control, a human placental DNA treated in vitro with excess of SssI methyltransferase that generates a completely methylated DNA. Ten microliters of PCR products were run on a 2% agarose gel and visualized by staining with ethidium bromide.

To evaluate the prevalence of methylation at CpG islands in the context of the sequence, MSP PCR products from all cell lines and from 5 randomly selected samples/each lesion cathegory (total n = 35) were subjected to direct cycle sequencing, using Big Dye Terminator Cycle Sequencing Kit chemistry (ABI PRISM Big Dye Terminator Cycle Sequencing Kit; PE Applied Biosystems, Warrington, United Kingdom) and were run on ABI PRISM 310 Genetic Analyzer (PE Applied Biosystems, Foster City, CA, USA).

### Real-Time MSP

Real-Time Methylation Specific PCR (MSP) was used for the quantification of the methylated and unmethylated *RASSF1A *promoters in all samples under study. Reactions were in triplicate for each individual sample.

Real-time PCR is based on the continuous optical monitoring of a fluorogenic PCR [27]. PCR amplifications were performed using DyNAmo HS SyBR Green qPCR Kit (Finnzymes) according to the manufacturer's instructions. SYBR Green I is specific for double-stranded DNA and fluoresces when bound to the amplified double-stranded PCR product, thereby enabling direct quantification of amplified DNA without labeled probes [28].

**Figure 1:**
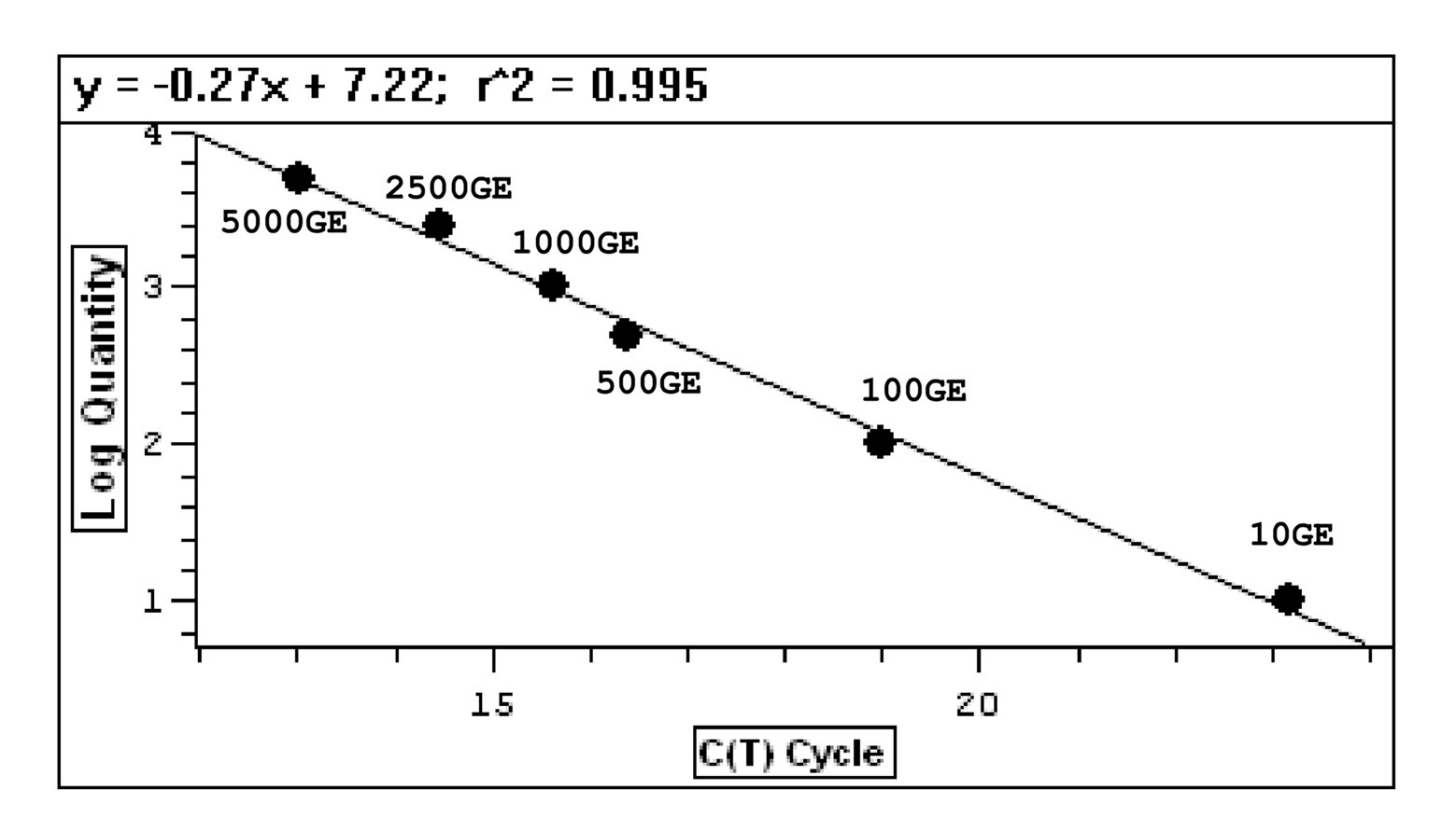
*RASSF1A *calibration curve of methylated DNA (LoVo): plot of Ct against the input target quantity (common logarithmic scale). The input target quantity is expressed as genome equivalents of LoVo DNA. The correlation cofficient is 0.995.

Amplifications were carried out in 96-well plates in an Chromo 4 thermalcycler (MJ Research Inc.), with 0.2 μM primers specific to methylated or unmethylated bisulfite-modified template and 50 ng of modified DNA in a 20 μl final reaction volume. The primers used for RASSF1A real-time MSP were the same used for conventional MSP (Table 2). Thermal cycling was initiated with a incubation step of 15 min at 95°C, followed by 45 cycles (95°C for 15 sec, 55°C for 20 sec, 72°C for 20 sec) with a single fluorescent reading taken at the end of each cycle. Each run was completed with a melting curve analysis to confirm the specificity of amplification and lack of primer dimers. Threshold cycle values, C_t_, were determined by the Opticon2 software. A calibration curve was run in parallel with each analysis. Human cell lines, LoVo and HeLa, previously shown to be respectively *RASSF1A *emimethylated and unmethylated by conventional MSP, were used for constructing the calibration curve for the RASSF1A M and U real-time MSP (correlation coefficient were: r = 0.992 ± 0,007 for M and r = 0,997 ± 0,003 for U). To set-up the calibration curve, we prepared serial dilutions of LoVo and HeLa DNA, containing 10, 100, 500, 1000, 2500 and 5000 genome-equivalents, one genome-equivalent being the amount of a particular target sequence in a single reference cell (Figure 1). A conversion factor of 6.6 pg of DNA per diploid cell was used for expressing quantitative results in genome equivalents [29,30].

The methylation index (%) in a sample was calculated according to the following equation:

Methylation index=MM+U×100
 MathType@MTEF@5@5@+=feaafiart1ev1aaatCvAUfKttLearuWrP9MDH5MBPbIqV92AaeXatLxBI9gBaebbnrfifHhDYfgasaacH8akY=wiFfYdH8Gipec8Eeeu0xXdbba9frFj0=OqFfea0dXdd9vqai=hGuQ8kuc9pgc9s8qqaq=dirpe0xb9q8qiLsFr0=vr0=vr0dc8meaabaqaciaacaGaaeqabaqabeGadaaakeaacqqGnbqtcqqGLbqzcqqG0baDcqqGObaAcqqG5bqEcqqGSbaBcqqGHbqycqqG0baDcqqGPbqAcqqGVbWBcqqGUbGBcqqGGaaicqqGPbqAcqqGUbGBcqqGKbazcqqGLbqzcqqG4baEcqGH9aqpdaWcaaqaaiabb2eanbqaaiabb2eanjabgUcaRiabbwfavbaacqGHxdaTcqaIXaqmcqaIWaamcqaIWaamaaa@4D7D@

where *M *and *U *are genome-equivalents of methylated and unmethylated *RASSF1A *sequences obtained by the interpolation of Ct values with M and U calibration curves.

A representative set of the original graphs (n = 12) from each group of lesions occurring in both hepatitic and non hepatitic liver is reported [see Additional file 1 and 2].

### Statistical analysis

Differences in the degree of methylation among categories of lesions were analyzed by linear mixed models (random effect model) accounting for the within subject correlations. Statistical association between age and methylation index were analyzed by Pearson correlation. p < 0.05 was considered to be statistically significant. Statistical analysis were performed by using STATA 9 software.

## Results

### RASSF1A promoter methylation in hepatitic liver

#### Qualitative analysis for the presence of methylated alleles by MSP

**Figure 2:**
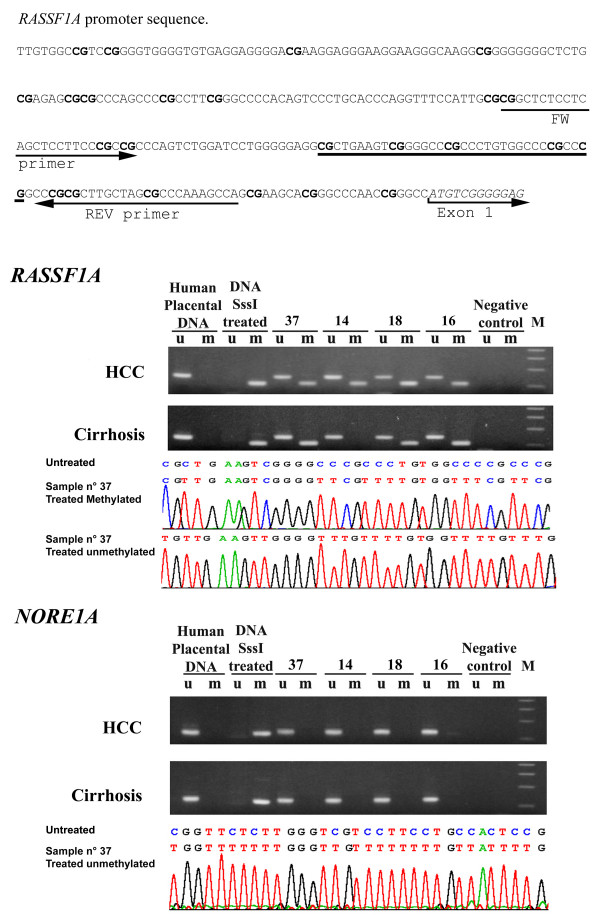
Upper panel: *RASSF1A *promoter region with CpG islands subjected to analysis; underlined is the sequence reported in the middle panel showing the methylation profile of *RASSF1A *detected by MSP in HCC and corresponding cirrhosis. Middle panel: Methylation profile of *RASSF1A *detected by MSP in HCC and corresponding cirrhosis of cases 14,16,18 and 37. DNASssI treated: positive control for methylation; M: molecular marker; Human Placental DNA: negative control for methylation; m: methylated; u: unmethylated. The illustrated sequence refers to a HCC (case n = 37) showing both methylated and unmethylated alleles. Lower panel: Methylation profile of *NORE1A *detected by MSP in HCC and corresponding cirrhosis of cases 14,16,18 and 37. DNASssI treated: positive control for methylation; M: molecular marker; Human Placental DNA: negative control for methylation; m: methylated; u: unmethylated. The illustrated sequence refers to a HCC (case n = 37) showing the exclusive presence of unmethylated alleles.

*RASSF1A *promoter methylation was detected by conventional MSP, in 100% (26/26) HCC, 86.6% (39/45) HN and 81.1% (30/37) cirrhotic livers. Figure 2 (upper panel) shows the methylation pattern of conventional MSP in cases of HCC and paired cirrhosis. HCC cell lines, but not the cell line Chang, were also methylated. DNA sequencing indicates that for methylated alleles, all (5/5; 100%) CpG sites in the context of the analyzed sequence (Figure 2) were resistant to bisulfite conversion, whereas all cytosine at non-CpG (17/17, 100%) sites were converted to thymidine residues (Figure 2). Conversely unmethylated samples never showed any resistant-to-bisulphite-conversion CpG site. No correlation was found between *RASSF1A *gene promoter methylation with the etiology of cirrhosis.

#### Quantitative analysis for the prevalence of methylated alleles by Real Time MSP

The calculated methylation indices for cell lines were as follows: HepG2 99.53%, Plc/Prf/5 100%, Huh7 100%, Huh6 100%, Chang 0.12%.

**Figure 3:**
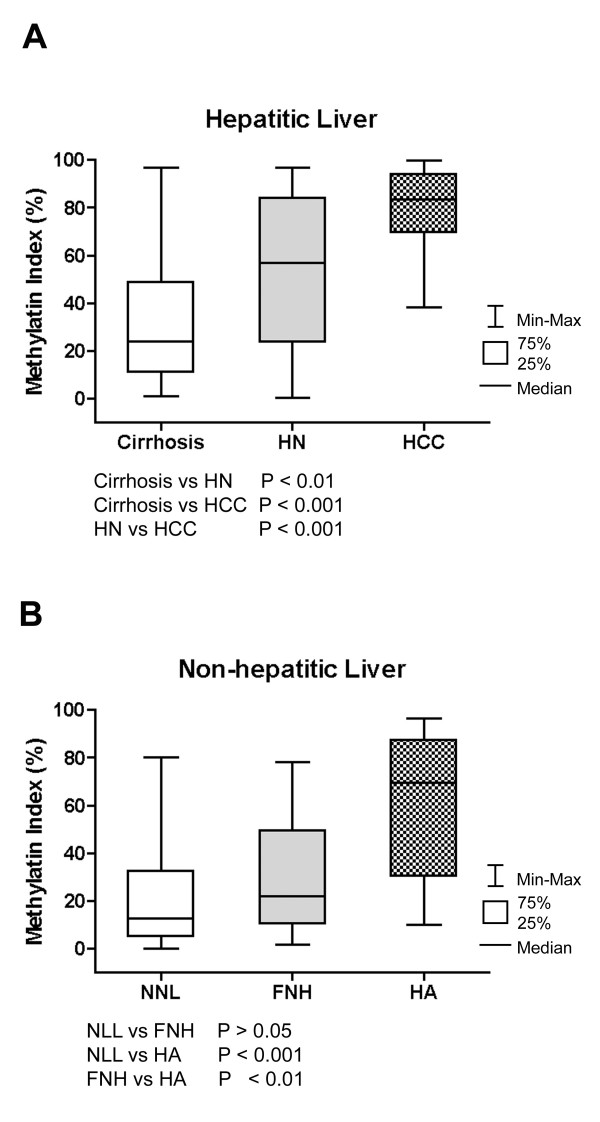
Quantitative evaluation of *RASSF1A *methylation by Real-Time MSP. The median value of methylation index, 25 and 75% percentiles, and minimum ad maximum values detected in liver cirrhosis, hepatocellular nodules (HN) and hepatocellular carcinoma (HCC) is reported in panel A; the median value of methylation index, 25 and 75% percentiles, and minimum and maximum values detected in non-lesional liver (NLL), focal nodular hyperplasia (FNH) and hepatocellular adenoma (HA) is reported in panel B.

A gradual but significant increase in the methylation index of *RASSF1A *was seen from cirrhosis to HN to HCC (Figure 3A). Cirrhotic tissue had a methylation index lower than that detected in HN (p < 0.01) and the latter a methylation index significantly lower than that detected in HCC (p < 0.001). HN were grouped together because no statistical difference was seen as to the methylation index among LGDN, HGDN and LRN (data not shown). No correlation was found between *RASSF1A *methylation index with the etiology of cirrhosis.

### RASSF1A promoter methylation in non-hepatitic liver

#### Qualitative analysis for the presence of methylated alleles by MSP

*RASSF1A *promoter methylation was detected by conventional MSP, in 100% (5/5) HA, 87.5% (7/8) FNH and 84.6% (11/13) non-lesional liver.

#### Quantitative analysis for the prevalence of methylated alleles by Real Time MSP

Hepatocellular adenomas (HA) showed a methylation index significantly higher than that detected in focal nodular hyperplasia (FNH) (p < 0.01) and in non-lesional tissue (p < 0.001) (Figure 3B). As shown in Table 3 a certain degree of inter-sample heterogeneity was seen in methylation index in individual cases of non-lesional liver, FNH and HA with multiple sampling. Specifically, a methylation index < 10% was detectable in 7/13 cases (15/34 samples) of non-lesional liver, 2/8 cases (4/22 samples) of FNH but never in HA. By contrast a methylation index > 50% was seen in 2/13 cases (2/34 samples) of non-lesional liver, 2/8 cases (3/22 samples) of FNH and 3/5 cases (7/13 samples) of HA.

**Figure 4:**
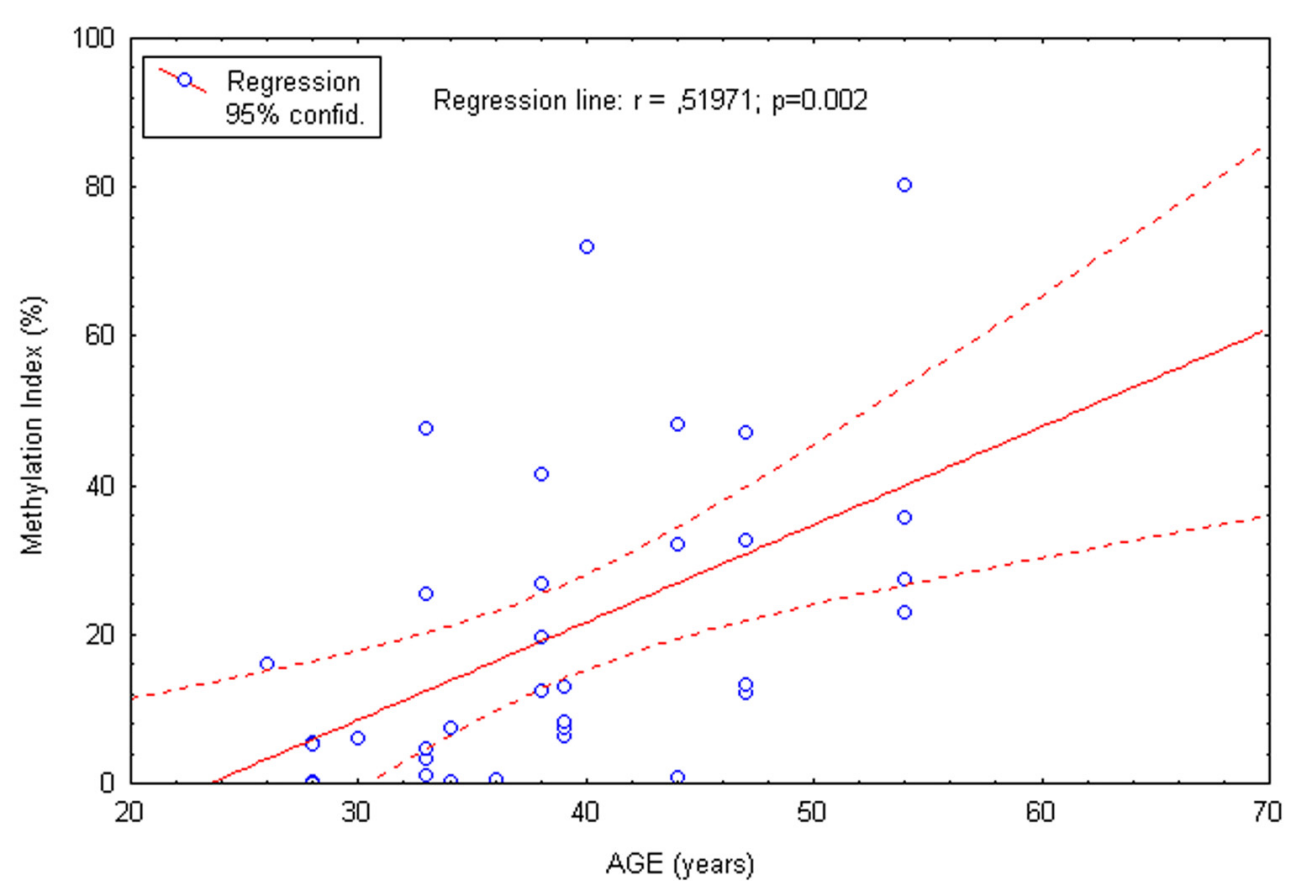
Non-lesional liver: methylation index of RASSF1A according to age.

In non-lesional liver, a gradual increase was seen in the methylation index by ageing (p = 0.002) (Figure 4).

### NORE1A gene promoter methylation in hepatitic and non-hepatitic liver

In all samples under study *NORE1A *gene was always found unmethylated by conventional MSP (Figure 2; lower panel).

## Discussion

We investigated the methylation profile of *RASSF1A *and *NORE1A *promoter genes, in non-lesional, cirrhotic, hyperplastic, dysplastic and neoplastic (benign and malignant) hepatocellular growths. These two genes are functionally thought to act in concert to mediate proapoptotic signal on the RAS pathway *in vivo *[31]. Given that RAS gene is not alterated in human hepatocarcinogenesis, we sought to analyze by MSP if these RAS effectors are epigenetically altered by promoter methylation in the above conditions. In keeping with several authors [13-16] we observed high frequency of *RASSF1A *methylation in HCC (100%) and associated cirrhosis (81.1%). In addition we observed a high RASSF1A methylation frequency (86.6%) in hepatocellular dysplastic nodules.

Interestingly, the *RASSF1A *methylation rate in cirrhosis (81–82%) and corresponding HCC (98–100%), as detected by MSP (qualitative assay), occured with a surprisingly overlapping frequency in 3 different geographic regions: Italy (mediterranean area, present work), North Europe [32] and China [14]. Given the different etiology of chronic liver disease in these regions, the above results might suggest that the very high rate of *RASSF1A *methylation in the cirrhosis-HCC sequence is a peculiar early molecular feature of hepatocarcinogenesis in the cirrhotic setting, unrelated to a specific etiology.

Lee et al. [17] reported 67% promoter methylation in HCC, 10% in dysplastic nodules and no methylation in non neoplastic hepatitic/cirrhotic liver. This discrepancy might be due to the sensitivity of our method (nested MSP), relying on the analysis of a different set of CpGs. Indeed Lee et al. [17] designed a set of primers targeted to amplify a sequence of exon 1 of the gene and not of its promoter, as we did.

Opposite results to *RASSF1A*, were obtained by analyzing *NORE1A *promoter methylation, which was not investigated previously in the human liver. This gene, which is supposed to act in concert with *RASSF1A *as proapoptotic mediator of RAS signalling, was never found methylated in our series of non-lesional, cirrhotic, hyperplastic, dysplastic and neoplastic liver. Hesson et al. [24] reported that this gene was variably methylated in a number of different human tumors such as breast, pulmonary, kidney and colorectal. In the present study, the unmethylation of *NORE1A *in the liver might be related to opposite epigenetic alteration of *RASSF1A*. According to this hypothesis Hesson et al. [33] recently reported frequent *RASSF1A*, but not *NORE1A*, methylation in gliomas. Given that both genes act in concert, it can be speculated that the methylation of *NORE1A *could result biologically redundant.

Based on these results we performed a *RASSF1A *quantitative analysis of methylation by Real-Time PCR on non-lesional, cirrhotic, hyperplastic, neoplastic liver and for the first time on dysplastic hepatocellular nodules.

In hepatitic livers, our quantitative analysis showed that HCC, as compared to cirrhosis, had a significantly higher methylation index of *RASSF1A*. This result is in keeping with that of Zhong et al. [15] measuring the number of methylated hepatocytes in HCC and cirrhosis by PCR-RFLP. Interestingly, in our series, quantitation of *RASSF1A *methylation showed that hepatocellular nodules had a methylation index in between cirrhosis and HCC, in keeping with their putative HCC precursor role.

The methylation of *RASSF1A *gene promoter, already set in our cases of cirrhosis, also supports a tumor-independent mechanism of gene methylation. Waki et al [20] recently showed, in an autopsy study, that all patients over 40 yrs had the *RASSF1A *gene promoter methylated in the liver, suggesting an age-related mechanism of gene methylation.

To probe whether *RASSF1A *gene promoter was also methylated in connection to age we included in our series, non-lesional liver samples taken far from lesion in patients with FNH or HA.

In the non-lesional liver, one or more samples of 5 out of 9 patients under 40 yrs, showed a methylation index grater than 10%. Interestingly, methylation index in non-lesional liver showed a gradual increase with ageing, that started before 30 years [32]. These results suggest that age-related methylation of *RASSF1A *gene promoter takes place early in a small subpopulation of cells of the human liver and that this fraction of hepatocytes is fated to increase by ageing. This observation resembles that reported by Nakagawa et al. [34] as to the methylation pattern of *hMLH1 *in the human colon, where methylated cells are distributed at multiple sites in the colon and methylation increased with age. Conceivably the fraction of hepatocytes already methylated with age at the *RASSF1A *gene promoter, might become, over time, more susceptible to oncogenic injuries. Because our results were obtained in a limited number of cases, the gradual increase of *RASSF1A *methylation index by age should be confirmed in larger series including younger subjects such as children and fetuses. As detected between HCC (a neoplastic growth) and cirrhosis (a regenerative growth), we also observed a statistically significant increase in the methylation index between hepatocellular adenoma (a neoplastic growth) and focal nodular hyperplasia (a hyperplastic growth). It has been suggested by Toyota et al. [21] that age-related methylation by potentially affecting genes that regulate both cell growth and differentiation could account in part for the hyperproliferative state that precedes tumor formation, thus linking age-related methylation to cancer-related methylation. The analysis of *RASSF1A *methylation index in the whole series of cases showed a gradual increase in the methylation index from non-lesional liver to regenerative/hyperplastic conditions (chronic liver disease and FNH), to preneoplastic lesions (HN) to overt tumors (HA and HCC). This result suggests that quantitative analysis of *RASSF1A *gene promoter methylation, rather than the detection of methylation bands "*per se*", might be clinically relevant. Quantitative results should also be compared to expression data (not available in the present series given the lack of specific antibodies and the unavailability of frozen material for mRNA analysis), to document the functional role of methylation.

## Conclusion

In conclusion we have shown that in non-lesional, cirrhotic, hyperplastic, dysplastic and neoplastic liver growths the *RASSF1A *gene is methylated, that this condition takes place as an age-related phenomenon and that the early setting and spreading over time of an epigenetically alterated hepatocyte subpopulation, might be related to liver tumorigenesis.

## Abbreviations

HCC: Hepatocellular Carcinoma; HA: Hepatocellular Adenoma; FNH: Focal Nodular Hyperplasia; HN: Hepatocellular Nodules; HGDN: High-Grade Dysplastic Nodule; LGDN: Low-Grade Dysplastic Nodule; LRN: Large Regenerative Nodule; NLL: Non-Lesional Liver.

## Competing interests

The author(s) declare that they have no competing interests.

## Authors' contributions

SDG: main banch investigator; AD, PB: banch work; LL: clinical information; FG: data elaboration; AMal: clinical information; ML: cell lines provider; AMor: statistical analysis; MR: coordinator, manuscript preparation. All authors read and approved the final manuscript.

## Pre-publication history

The pre-publication history for this paper can be accessed here:



## Supplementary Material

Additional File 1 **Real-Time PCR experiments of hepatitic liver. **A representative set of the original graphs of Real-Time PCR experiments of lesions occurring in the hepatitic liver (Cirrhosis, Hepatocellular Nodules and HCC). (file format: JPEG) Click here for file

Additional File 2 **Real-Time PCR experiments of non-hepatitic liver. **A representative set of the original graphs of Real-Time PCR experiments of lesions occurring in non-hepatitic liver (non lesional liver, focal nodular hyperplasia and hepatocellular adenoma). (file format: JPEG) Click here for file

## Figures and Tables

**Table 1:** Clinico-pathological features of the series. Numbers in columns refer to the number of individual lesions and to the number of tissue samples (parenthesis).

**Pts No**	**Age**	**Sex**	**Etiology**	**HCC n = 26 (30)**	HN n = 45 (45)	**HA n = 5 (13)**	**FNH n = 8 (22)**	**Cirrhosis n = 37 (39)**	**NLL n = 13 (34)**
**Hepatitic Livers**									
***1***	70	M	HBV	1 (1)	--	--	--	1 (1)	--
***2***	48	M	HBV	1 (1)	2 (2)	--	--	1 (1)	--
***3***	50	M	HBV	--	2 (2)	--	--	1 (1)	--
***4***	41	M	HBV	--	1 (1)	--	--	1 (1)	--
***5***	41	M	HBV	--	3 (3)	--	--	1 (1)	--
***6***	59	M	HCV	--	2 (2)	--	--	1 (1)	--
***7***	56	M	HCV	--	1 (1)	--	--	1 (1)	--
***8***	58	M	HCV	--	1 (1)	--	--	1 (1)	--
***9***	75	F	HCV	1 (4)	3 (3)	--	--	1 (1)	--
***10***	42	M	HCV	1 (1)	2 (2)	--	--	1 (1)	--
***11***	59	F	HCV	1 (2)	1 (1)	--	--	1 (1)	--
***12***	71	F	HCV	1 (1)	2 (2)	--	--	1 (1)	--
***13***	71	F	HCV	1 (1)	3 (3)	--	--	1 (2)	--
***14***	57	M	HCV	1 (1)	--	--	--	1 (1)	--
***15***	62	F	HCV	1 (1)	--	--	--	1 (1)	--
***16***	67	M	HCV	1 (1)	--	--	--	1 (1)	--
***17***	74	M	HCV	1 (1)	--	--	--	1 (1)	--
***18***	67	M	HCV	1 (1)	--	--	--	1 (1)	--
***19***	70	F	HCV	1 (1)	--	--	--	1 (1)	--
***20***	69	M	HCV	1 (1)	--	--	--	1 (1)	--
***21***	68	F	HCV	1 (1)	--	--	--	1 (1)	--
***22***	54	M	HBV/HCV	--	2 (2)	--	--	1 (1)	--
***23***	49	M	HBV/HDV	1 (1)	1 (1)	--	--	1 (1)	--
***24***	53	M	HBV/HDV	--	3 (3)	--	--	1 (1)	--
***25***	52	F	HBV/HDV	--	4 (4)	--	--	1 (1)	--
***26***	62	M	HBV/HDV/HCV	1 (1)	5 (5)	--	--	--	--
***27***	65	M	Cryptogenic	1 (1)	--	--	--	1 (1)	--
***28***	48	F	Cryptogenic	1 (1)	--	--	--	1 (1)	--
***29***	72	M	Cryptogenic	1 (1)	--	--	--	1 (1)	--
***30***	57	M	Cryptogenic	--	2 (2)	--	--	1 (1)	--
***31***	51	F	Cryptogenic	1 (1)	--	--	--	1 (1)	--
***32***	73	M	Cryptogenic	1 (1)	--	--	--	1 (1)	--
***33***	70	F	Cryptogenic	1 (1)	--	--	--	1 (1)	--
***34***	49	M	Cryptogenic	--	3 (3)	--	--	1 (2)	--
***35***	69	M	Cryptogenic	1 (1)	1 (1)	--	--	1 (1)	--
***36***	22	F	Wilson desease	--	1 (1)	--	--	1 (1)	--
***37***	65	M	Alcholic	1 (1)	--	--	--	1 (1)	--
***38***	70	M	Haemocromatosis	1 (1)	--	--	--	1 (1)	--
**Non-Hepatitic Livers**									
***39***	28	M	--	--	--	--	1 (3)	--	1 (4)
***40***	30	F	--	--	--	--	1 (1)	--	1 (1)
***41***	39	M	--	--	--	--	1 (4)	--	1 (4)
***42***	47	F	--	--	--	--	1 (3)	--	1 (4)
***43***	26	F	--	--	--	--	1 (1)	--	1 (1)
***44***	33	M	--	--	--	--	1 (5)	--	1 (4)
***45***	54	M	--	--	--	--	1 (4)	--	1 (4)
***46***	33	F	--	--	--	--	1 (1)	--	1 (1)
***47***	44	F	--	--	--	1 (4)	--	--	1 (3)
***48***	36	F	--	--	--	1 (1)	--	--	1 (1)
***49***	34	F	--	--	--	1 (3)	--	--	1 (2)
***50***	38	F	--	--	--	1 (4)*	--	--	1 (4)
***51***	40	F	--	--	--	1 (1)	--	--	1 (1)

HCC = hepatocellular carcinoma; HN = hepatocellular nodules; HA = hepatocellular adenoma; FNH = focal nodular hyperplasia; NLL = non-lesional liver.

* Multiple adenomatosis.

**Table 2:** Primers used in this study

***Primer***	***Forward***	***Reverse***
*RASSF1A mod*	GGGTTTTATAGTTTTTGTATTTAGGTT	AACTCAATAAACTCAAACTCCCC
*RASSF1A met*	CGGTTTTTTTTAGTTTTTTTTCGTCG	TAACTTTAAACGCTAACAAACGCGAA
*RASSF1A unmet*	TGTGTGGTTTTTTTTAGTTTTTTTTTGTTG	CCCAACATAACCCAATTAAACCCA
*NORE1A mod*	GAATTTTGTAGTTGTTTTAGGTGAAGA	CTACTCCCR*AACCAACCCC
*NORE1A met*	TATTCGCGTAGACGTCGTTTGGTAC	AAACCGCAACCTACTAAACGAAACG
*NORE1A unmet*	ATTTATATTTGTGTAGATGTTGTTTGGTATGGA	TTTAAAAAAACCACAACCTACTAAACAAAACA

*R = G or A

**Table 3:** *RASSF1A *methylation index in hepatocellular adenoma (HA), focal nodular hyperplasia (FNH) and corresponding non-lesional liver (NLL) with viable multiple samplings. Each dot refers to *RASSF1A *methylation index (%) in one sampling. In brackets: number of tissue samples analyzed in each group of lesions.

***Patient***	Age	HA (n = 13)				***FNH ***(n = 22)					***NLL ***(n = 34)			
***43***	**26**													
***39***	**28**										○	○	○	○
***40***	**30**													○
***46***	**33**													
***44***	**33**									○		○	○	○
***49***	**34**		●	●									○	○
***48***	**36**													○
***50***	**38**													
***41***	**39**							○	○	○		○	○	○
***51***	**40**				●									●
***47***	**44**	●	●	●	●									○
***42***	**47**							●						
***45***	**54**						●	●			●			

○ = *RASSF1A *methylation index: <10%

= *RASSF1A *methylation index: 10–50%

● = *RASSF1A *methylation index: >50%

## References

[B1] Lerman MI, Minna JD (2000). The 630-kb lung cancer homozygous deletion region on human chromosome 3p21.3: identification and evaluation of the resident candidate tumor suppressor genes. The International Lung Cancer Chromosome 3p21.3 Tumor Suppressor Gene Consortium. Cancer Res.

[B2] Dammann R, Li C, Yoon JH, Chin PL, Bates S, Pfeifer GP (2000). Epigenetic inactivation of a RAS association domain family protein from the lung tumour suppressor locus 3p21.3. Nat Genet.

[B3] Agathanggelou A, Honorio S, Macartney DP, Martinez A, Dallol A, Rader J, Fullwood P, Chauhan A, Walker R, Shaw JA, Hosoe S, Lerman MI, Minna JD, Maher ER, Latif F (2001). Methylation associated inactivation of RASSF1A from region 3p21.3 in lung, breast and ovarian tumours. Oncogene.

[B4] Burbee DG, Forgacs E, Zochbauer-Muller S, Shivakumar L, Fong K, Gao B, Randle D, Kondo M, Virmani A, Bader S, Sekido Y, Latif F, Milchgrub S, Toyooka S, Gazdar AF, Lerman MI, Zabarovsky E, White M, Minna JD (2001). Epigenetic inactivation of RASSF1A in lung and breast cancers and malignant phenotype suppression. J Natl Cancer Inst.

[B5] Dammann R, Takahashi T, Pfeifer GP (2001). The CpG island of the novel tumor suppressor gene RASSF1A is intensely methylated in primary small cell lung carcinomas. Oncogene.

[B6] Dreijerink K, Braga E, Kuzmin I, Geil L, Duh FM, Angeloni D, Zbar B, Lerman MI, Stanbridge EJ, Minna JD, Protopopov A, Li J, Kashuba V, Klein G, Zabarovsky ER (2001). The candidate tumor suppressor gene, RASSF1A, from human chromosome 3p21.3 is involved in kidney tumorigenesis. Proc Natl Acad Sci U S A.

[B7] Lee MG, Kim HY, Byun DS, Lee SJ, Lee CH, Kim JI, Chang SG, Chi SG (2001). Frequent epigenetic inactivation of RASSF1A in human bladder carcinoma. Cancer Res.

[B8] Lo KW, Kwong J, Hui AB, Chan SY, To KF, Chan AS, Chow LS, Teo PM, Johnson PJ, Huang DP (2001). High frequency of promoter hypermethylation of RASSF1A in nasopharyngeal carcinoma. Cancer Res.

[B9] Morrissey C, Martinez A, Zatyka M, Agathanggelou A, Honorio S, Astuti D, Morgan NV, Moch H, Richards FM, Kishida T, Yao M, Schraml P, Latif F, Maher ER (2001). Epigenetic inactivation of the RASSF1A 3p21.3 tumor suppressor gene in both clear cell and papillary renal cell carcinoma. Cancer Res.

[B10] Astuti D, Agathanggelou A, Honorio S, Dallol A, Martinsson T, Kogner P, Cummins C, Neumann HP, Voutilainen R, Dahia P, Eng C, Maher ER, Latif F (2001). RASSF1A promoter region CpG island hypermethylation in phaeochromocytomas and neuroblastoma tumours. Oncogene.

[B11] Maruyama R, Toyooka S, Toyooka KO, Harada K, Virmani AK, Zochbauer-Muller S, Farinas AJ, Vakar-Lopez F, Minna JD, Sagalowsky A, Czerniak B, Gazdar AF (2001). Aberrant promoter methylation profile of bladder cancer and its relationship to clinicopathological features. Cancer Res.

[B12] Pfeifer GP, Yoon JH, Liu L, Tommasi S, Wilczynski SP, Dammann R (2002). Methylation of the RASSF1A gene in human cancers. Biol Chem.

[B13] Zhang YJ, Ahsan H, Chen Y, Lunn RM, Wang LY, Chen SY, Lee PH, Chen CJ, Santella RM (2002). High frequency of promoter hypermethylation of RASSF1A and p16 and its relationship to aflatoxin B1-DNA adduct levels in human hepatocellular carcinoma. Mol Carcinog.

[B14] Yu J, Ni M, Xu J, Zhang H, Gao B, Gu J, Chen J, Zhang L, Wu M, Zhen S, Zhu J (2002). Methylation profiling of twenty promoter-CpG islands of genes which may contribute to hepatocellular carcinogenesis. BMC Cancer.

[B15] Zhong S, Yeo W, Tang MW, Wong N, Lai PB, Johnson PJ (2003). Intensive hypermethylation of the CpG island of Ras association domain family 1A in hepatitis B virus-associated hepatocellular carcinomas. Clin Cancer Res.

[B16] Schagdarsurengin U, Wilkens L, Steinemann D, Flemming P, Kreipe HH, Pfeifer GP, Schlegelberger B, Dammann R (2003). Frequent epigenetic inactivation of the RASSF1A gene in hepatocellular carcinoma. Oncogene.

[B17] Lee S, Lee HJ, Kim JH, Lee HS, Jang JJ, Kang GH (2003). Aberrant CpG island hypermethylation along multistep hepatocarcinogenesis. Am J Pathol.

[B18] Roncalli M, Bianchi P, Bruni B, Laghi L, Destro A, Di Gioia S, Gennari L, Tommasini M, Malesci A, Coggi G (2002). Methylation framework of cell cycle gene inhibitors in cirrhosis and associated hepatocellular carcinoma.. Hepatology.

[B19] Kaneto H, Sasaki S, Yamamoto H, Itoh F, Toyota M, Suzuki H, Ozeki I, Iwata N, Ohmura T, Satoh T, Karino Y, Toyota J, Satoh M, Endo T, Omata M, Imai K (2001). Detection of hypermethylation of the p16INK4A gene promoter in chronic hepatitis and cirrhosis associated with hepatitis B or C virus. Gut.

[B20] Waki T, Tamura G, Sato M, Motoyama T (2003). Age-related methylation of tumor suppressor and tumor-related genes: an analysis of autopsy samples. Oncogene.

[B21] Toyota M, Issa JP (1999). CpG island methylator phenotypes in aging and cancer. Semin Cancer Biol.

[B22] Ortiz-Vega S, Khokhlatchev A, Nedwidek M, Zhang XF, Dammann R, Pfeifer GP, Avruch J (2002). The putative tumor suppressor RASSF1A homodimerizes and heterodimerizes with the Ras-GTP binding protein Nore1. Oncogene.

[B23] Tommasi S, Dammann R, Jin SG, Zhang Xf XF, Avruch J, Pfeifer GP (2002). RASSF3 and NORE1: identification and cloning of two human homologues of the putative tumor suppressor gene RASSF1. Oncogene.

[B24] Hesson L, Dallol A, Minna JD, Maher ER, Latif F (2003). NORE1A, a homologue of RASSF1A tumour suppressor gene is inactivated in human cancers. Oncogene.

[B25] Destro A, Bianchi P, Alloisio M, Laghi L, Di Gioia S, Malesci A, Cariboni U, Gribaudi G, Bulfamante G, Marchetti A, Bosari S, Infante M, Ravasi G, Roncalli M (2004). K-ras and p16(INK4A)alterations in sputum of NSCLC patients and in heavy asymptomatic chronic smokers.. Lung Cancer.

[B26] Herman JG, Graff JR, Myohanen S, Nelkin BD, Baylin SB (1996). Methylation-specific PCR: a novel PCR assay for methylation status of CpG islands. Proc Natl Acad Sci U S A.

[B27] Heid CA, Stevens J, Livak KJ, Williams PM (1996). Real time quantitative PCR. Genome Res.

[B28] Rutledge RG, Cote C (2003). Mathematics of quantitative kinetic PCR and the application of standard curves. Nucleic Acids Res.

[B29] Saiki RK, Gelfand DH, Stoffel S, Scharf SJ, Higuchi R, Horn GT, Mullis KB, Erlich HA (1988). Primer-directed enzymatic amplification of DNA with a thermostable DNA polymerase. Science.

[B30] Lo YMD, Wong IHN, Zhang J, Tein MSC, Ng MHL, Hjelm NM (1999). Quantitative Analysis of Aberrant p16 Methylation Using Real-Time Quantitative Methylation-specific Polymerase Chain Reaction. Cancer Res.

[B31] Malumbres M, Barbacid M (2003). RAS oncogenes: the first 30 years. Nat Rev Cancer.

[B32] Lehmann U, Berg-Ribbe I, Wingen LU, Brakensiek K, Becker T, Klempnauer J, Schlegelberger B, Kreipe H, Flemming P (2005). Distinct Methylation Patterns of Benign and Malignant Liver Tumors Revealed by Quantitative Methylation Profiling. Clin Cancer Res.

[B33] Yu J, Ni M, Xu J, Zhang H, Gao B, Gu J, Chen J, Zhang L, Wu M, Zhen S, Zhu J (2002). Methylation profiling of twenty promoter-CpG islands of genes which may contribute to hepatocellular carcinogenesis.. BMC Cancer.

[B34] Hesson L, Bieche I, Krex D, Criniere E, Hoang-Xuan K, Maher ER, Latif F (2004). Frequent epigenetic inactivation of RASSF1A and BLU genes located within the critical 3p21.3 region in gliomas. Oncogene.

[B35] Nakagawa H, Nuovo GJ, Zervos EE, Martin EWJ, Salovaara R, Aaltonen LA, de la Chapelle A (2001). Age-related hypermethylation of the 5' region of MLH1 in normal colonic mucosa is associated with microsatellite-unstable colorectal cancer development. Cancer Res.

