# Supplementary figures and images for: Quantitative evaluation of RASSF1A methylation in the non-lesional, regenerative and neoplastic liver
Source: BMC Cancer. 2006 Apr 10;6:89. doi: 10.1186/1471-2407-6-89 (PMC1479360; doi:10.1186/1471-2407-6-89)

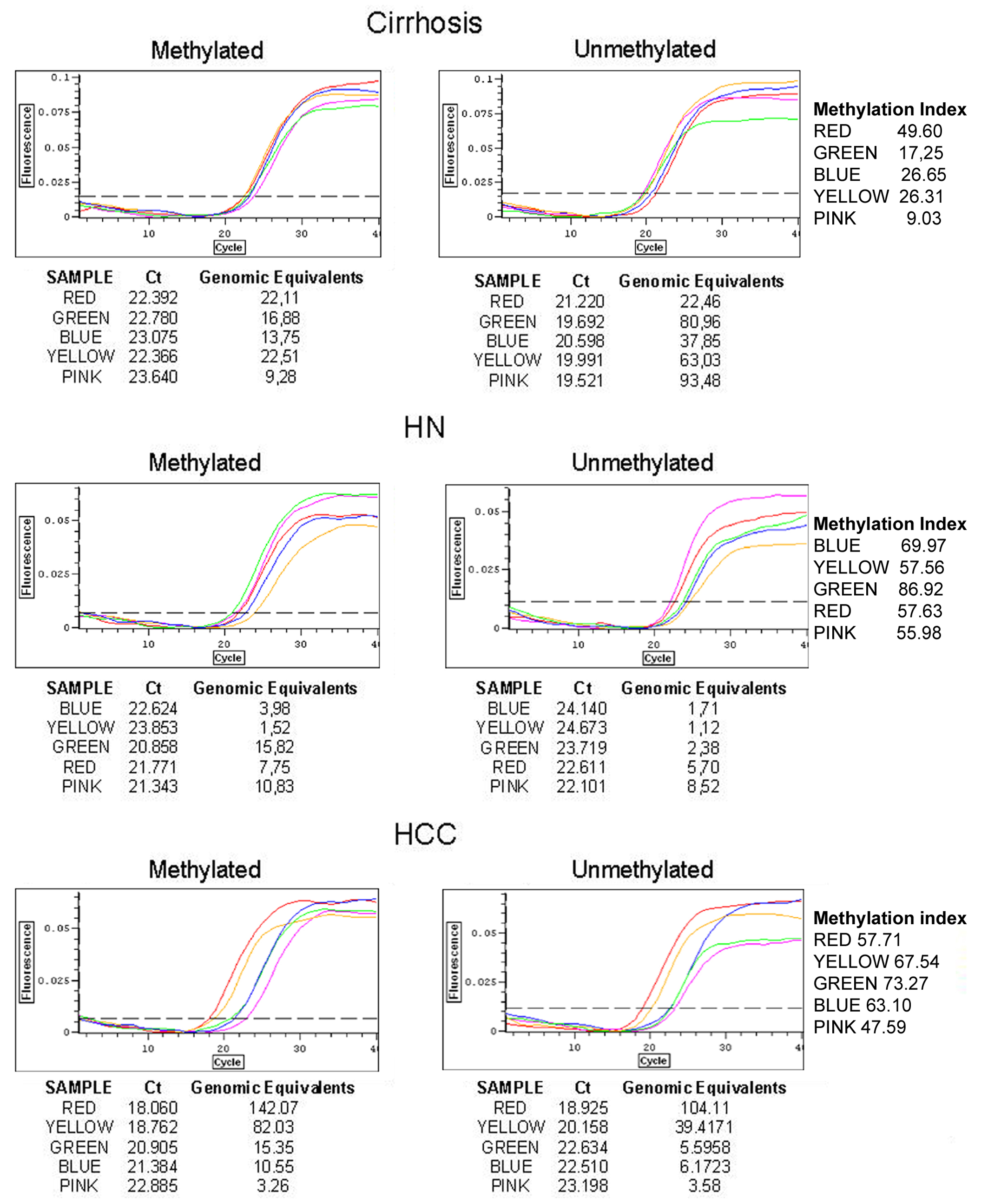

Supplement: Additional File 1 — Real-Time PCR experiments of hepatitic liver. A representative set of the original graphs of Real-Time PCR experiments of lesions occurring in the hepatitic liver (Cirrhosis, Hepatocellular Nodules and HCC). (file format: JPEG) [file 1471-2407-6-89-S1.jpeg]

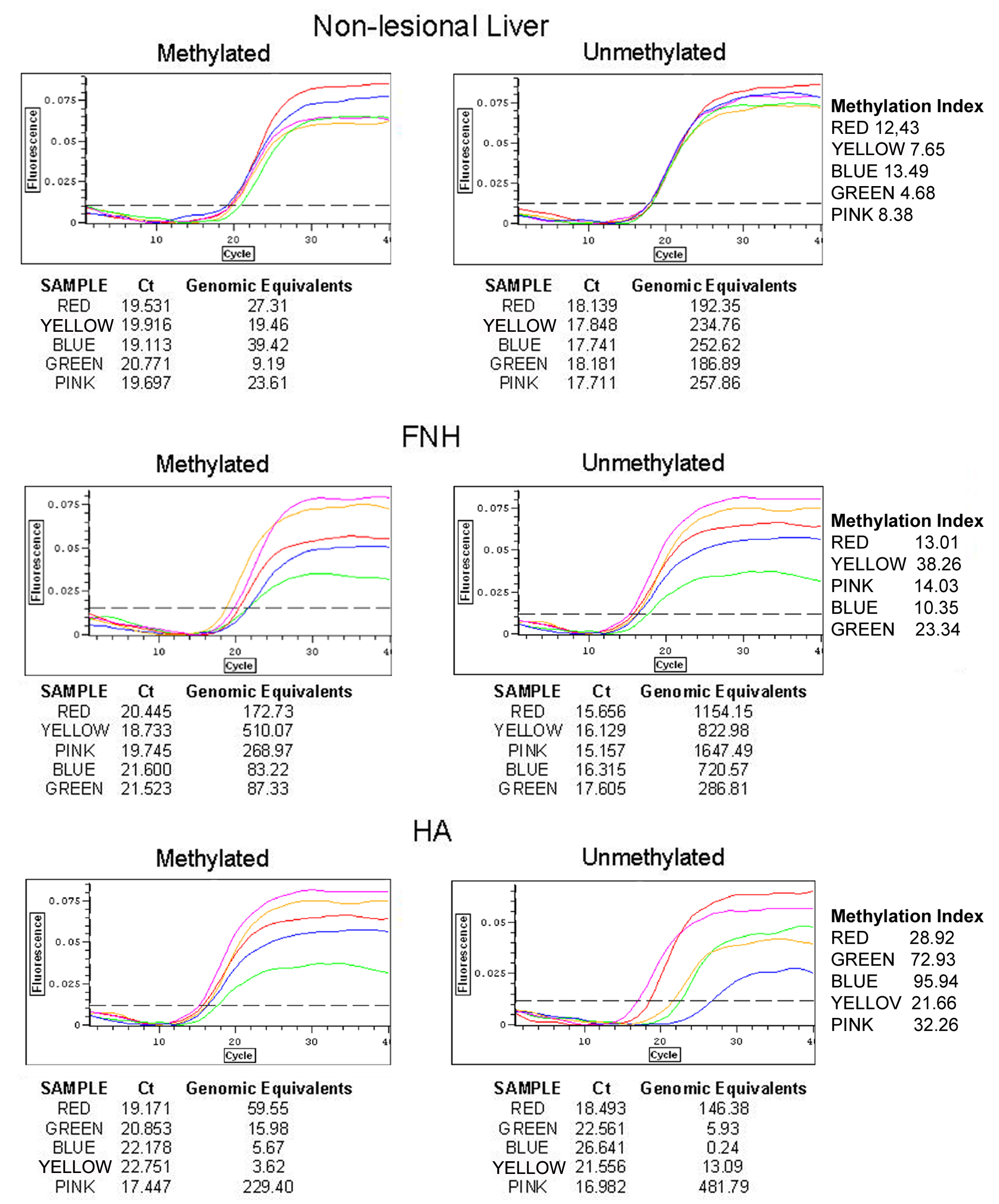

Supplement: Additional File 2 — Real-Time PCR experiments of non-hepatitic liver. A representative set of the original graphs of Real-Time PCR experiments of lesions occurring in non-hepatitic liver (non lesional liver, focal nodular hyperplasia and hepatocellular adenoma). (file format: JPEG) [file 1471-2407-6-89-S2.jpeg]
